# Retraction Note: Attenuating effect of Ginsenoside Rb1 on LPS-induced lung injury in rats

**DOI:** 10.1186/s12950-023-00368-5

**Published:** 2023-11-29

**Authors:** Qing Yuan, Yan-wen Jiang, Ting-ting Ma, Qiu-hong Fang, Lei Pan

**Affiliations:** 1 Intensive Care Unit of Geriatrics, Beijing Shijitan Hospital Affiliated to Capital Medicine University, No.10 Tieyi Road, Beijing, Haidian District 100038 People’s Republic of China; 2 Department of Pulmonary and Critical Care, Medicine, Beijing Shijitan Hospital Affiliated to Capital Medicine University, No.10 Tieyi Road, Beijing, Haidian District 100038 People’s Republic of China; 3 Department of Geriatrics, Beijing Shijitan Hospital Affiliated to Capital Medicine University, No.10 Tieyi Road, Beijing, Haidian District 100038 People’s Republic of China

The Editors-in-Chief have retracted this article. After publication, concerns were raising regarding extensive overlap between the data presented in this article and another article from the same authors that was under consideration within a similar time frame [1].

The authors have stated that the data were misused in the other article [1] by mistake and provided the raw data for validation. However, further checks by the Publisher found several cases of overlap in the raw data images representing different groups, as well as signs of image editing.

The Editors-in-Chief therefore no longer have confidence in the presented data.

Qing Yuan agrees to this retraction. Yan-wen Jiang, Ting-ting Ma and Qiu-hong Fang have not responded to any correspondence from the editor or publisher about this retraction. The Publisher has not been able to obtain a current email address for Lei Pan.

## References

[1] Yuan Q, Jiang YW, Fang QH (2014). Improving effect of Sivelestat on lipopolysaccharide-induced lung injury in rats. APMIS.

